# Reliable Industry 4.0 Based on Machine Learning and IoT for Analyzing, Monitoring, and Securing Smart Meters

**DOI:** 10.3390/s21020487

**Published:** 2021-01-12

**Authors:** Mahmoud Elsisi, Karar Mahmoud, Matti Lehtonen, Mohamed M. F. Darwish

**Affiliations:** 1 Industry 4.0 Implementation Center, Center for Cyber–Physical System Innovation, National Taiwan University of Science and Technology, Taipei 10607, Taiwan; mahmoud.elsisi@mail.ntust.edu.tw or; 2 Department of Electrical Engineering, Faculty of Engineering at Shoubra, Benha University, Cairo 11629, Egypt; 3 Department of Electrical Engineering and Automation, Aalto University, FI-00076 Espoo, Finland; karar.mostafa@aalto.fi (K.M.); matti.lehtonen@aalto.fi (M.L.); 4 Department of Electrical Engineering, Faculty of Engineering, Aswan University, Aswan 81542, Egypt

**Keywords:** smart systems, industry 4.0, internet of things, machine learning

## Abstract

The modern control infrastructure that manages and monitors the communication between the smart machines represents the most effective way to increase the efficiency of the industrial environment, such as smart grids. The cyber-physical systems utilize the embedded software and internet to connect and control the smart machines that are addressed by the internet of things (IoT). These cyber-physical systems are the basis of the fourth industrial revolution which is indexed by industry 4.0. In particular, industry 4.0 relies heavily on the IoT and smart sensors such as smart energy meters. The reliability and security represent the main challenges that face the industry 4.0 implementation. This paper introduces a new infrastructure based on machine learning to analyze and monitor the output data of the smart meters to investigate if this data is real data or fake. The fake data are due to the hacking and the inefficient meters. The industrial environment affects the efficiency of the meters by temperature, humidity, and noise signals. Furthermore, the proposed infrastructure validates the amount of data loss via communication channels and the internet connection. The decision tree is utilized as an effective machine learning algorithm to carry out both regression and classification for the meters’ data. The data monitoring is carried based on the industrial digital twins’ platform. The proposed infrastructure results provide a reliable and effective industrial decision that enhances the investments in industry 4.0.

## 1. Introduction

In recent years, updating market necessities and developing autonomous technologies, e.g., the internet of things (IoT), is shifting traditional power systems towards promising smart grids [1,2,3]. Typically, IoT is considered a system in which diverse physical components are enabled with embedded electronic systems (meters, sensors, etc.) while linking to the Internet [4,5,6]. The major benefit of IoT is its feature to seamlessly combine its physical components into the information network, thereby being active members in business processes while sharing information [6,7]. Several big data and machine learning approaches have been developed for different industrial applications, such as model-based vehicular prognostics framework [8] and health assessment [9]. Meanwhile, increasing carbon emissions and energy prices as well as raising awareness about energy efficiency are motivating decision-makers and power system planners to develop new efficient strategies to ensure optimal and secure power system operation. Interestingly, a promising area in which IoT could play a key role is smart meters, where it can enable the collection of electricity consumption data near real-time [10,11,12,13]. Such data are valuable for improving energy efficiency, where it can be integrated into energy production, management, and planning.

Electricity stealing and energy meter billing are considered the most important issues of the energy distribution system [14], where the usage of manual systems is unlikely. That is due to the different mistakes that occur by humans at different utility companies, which cause a problem for customers in the wrong reading. In addition, it is less accurate, less reliable, and non-tamper proof. Further, it is essential to enhance the accuracy of the billing system. To avoid human involvement in the process of billing, an automatic smart system for reading and transfer data of meters should be applied [15,16,17]. Presently, this technique is employed in the countries that can only monitor and measure the energy consumption of electricity, but do not permit remote access. The other problem with the current situation is that it needs a lot of manpower, which means time-consuming and various errors.

Smart energy meter can answer these problems by introducing various solutions and services to the consumer through transferring messages, including the power consumption (in KW), and when the credit is low, it automatically alerts to recharge money. Moreover, other beneficial features like tamper-proof, fault detection, etc., are developed. The numbers of these smart meters in power systems connected in the UK, the US, and China were 2900 thousand, 70,000 thousand, and 96,000 thousand respectively, in 2016 [18]. The execution of the smart meters will assist in better energy management evaluation, conservation of energy, and also indeed the needless disturbances of wrong billing, where the billing system is important in evaluating the pathway of consumption and resolving any dissimilarities on consumption and billing [19,20]. Smart meters and communication networks establish the advanced metering infrastructure (AMI), which can save the demand profiles and ease bi-directional data flow [21]. The smart energy meter has various merits like detecting faults in the distribution network by examining the supply status at a power transformer [22,23]. Indeed, these smart meters are considered the future vision that has massive advantages for not only consumers but also producers/suppliers.

The Fourth Industrial Revolution (i.e., Industry 4.0) and industrial IoT technologies are speedily driving data and software solutions powered digitalization in several subjects. Among the numerous advantages offered by them is the infrastructure for utilizing big data, machine learning, as well as cloud computing software platforms. Industry 4.0 is defined by the continuing automation of conventional manufacturing and industrial procedures, through diverse modern intelligent technologies. Specifically, large-scale machine-to-machine communication (M2M) and the IoT are combined for improved automation processes, superior communication and self-monitoring, and assembly of intelligent machines that can solve issues without the requirement of human interference [24,25]. In this work, we focus on the security part and data loss in the infrastructure of industry 4.0 and apply this topology in the smart meters, which can be applied in other smart sensors in the future, not the total industry 4.0 system for managing and securing smart meters.

As shown above, the recent control infrastructure of smart grids represents the most effective way to increase the efficiency of the grid. Specifically, it manages and monitors the communication between the smart machines (i.e., smart meters). For these reasons, this paper introduces an efficient infrastructure based on machine learning to analyze and monitor the data of smart meters. Further, it has the ability to investigate if this data is real data or fake, where fake data can be caused by the hacking of these meters. The merit of the proposed infrastructure is the consideration of the cyber-physical systems that are the basis of the fourth industrial revolution (labeled industry 4.0). Such adopted industry 4.0 involves the IoT and smart sensors (smart energy meters). Also, the proposed infrastructure can validate the amount of data loss through communication channels and the internet connection. A remarkable advantage is mitigating the industrial environment impacts on the efficiency of the meters (temperature, humidity, and noise signals). To do so, the decision tree is utilized in the proposed infrastructure as an effective machine learning algorithm to carry out both regression and classification for the meters’ data. Accordingly, the proposed infrastructure can provide a reliable and effective industrial decision that improves the investments in industry 4.0.

## 2. Architecture Description of the Proposed Infrastructure

**Figure 1:**
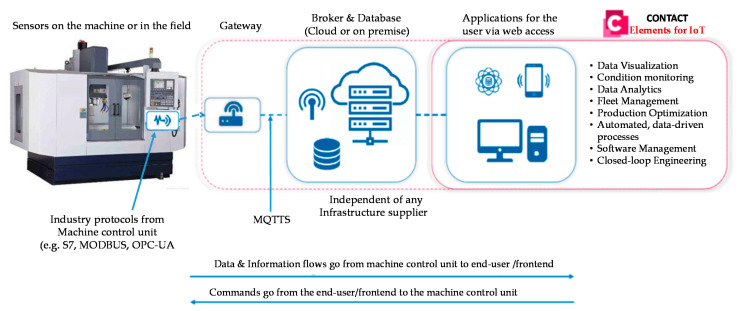
The schematic diagram of the infrastructure of Internet of Things (IoT) processing.

Today, a lot of industrial vendors provide computing services including hardware and software tools for IoT purposes. These services provide data analytics platforms for different industrial applications, as shown in Figure 1 [26]. The data acquisition process is performed by the measurements from the local sensors for different purposes such as storage, visualization, and analysis. The data acquisition is carried out by utilizing interfaces such as Modbus, Open Platform Communications (OPC) and different network protocols like Hypertext Transfer Protocol (HTTP) and Message Queue Telemetry Transport (MQTT). Currently, many IoT platforms are provided for data collection within edge computing devices and cloud gateway. The selection of the data acquisition process influences real-time monitoring. In the case of a high-latency manner, offline data analysis is preferred. Data visualization, selecting datasets, and data filtering are the popular common procedures for data pre-processing. In addition, the delay estimation, reduction of dimension, and resampling can be included in the data pre-processing. Then, the selection of the proper model including the training, testing, and validation data is an important stage for data analysis. The data analysis model depends on the environmental characteristics of each factory. The proposed system is carried out based on edge computing devices as realistic and parallel computing architecture. Each device collects the data from the machines and sends this data to the database server to overcome the different communication protocols of the sensors. The gateway classifies the collected data based on the Artificial Intelligence (AI) techniques and sends the final results via MQTT protocol to monitor it in the dashboard of the industrial digital twins’ platform.

The benefits of IoT integrated into the industry 4.0 is to enable real-time data interchange among a large variety of smart meters for different applicants, such as factories, homes, and hospitals, which are essential for smart grids. Indeed, the progress of IoT-enabled smart meters is empowered via a distinct network connection, e.g., Bluetooth and Global System for Mobiles (GSM), for examining and managing smart meters remotely. With the development in industry 4.0, the smart meter industry has used technologies like IoT and big data that allow incremental datasets from users and smart devices. Consequently, several frameworks and platforms are donated to specific industries and appliances using diverse technologies beneath the industry 4.0 umbrella. In this study, the advantage of utilizing the IoT framework under industry 4.0 for smart meters is that it enables an enterprise resource planning system, which can promote the manufactured electric industry in several features, including forecasting, real-time visibility, remote monitoring, cyber-physical security, and alerts and notifications.

## 3. Machine Learning Principles

Machine learning is divided into two categories, named supervised and unsupervised learning. Supervised learning means that the machine utilizes training data to learn what it should do. For example, if the process is to classify images of dogs and cats, the machine utilizes a labeled dataset about dog and cat images to learn the differences between the dogs and cats. Unsupervised learning is utilized to divided data groups into similar categories. For instance, if the inputs of the system are sets of cats and dogs’ images without any label of which is dog or cat, the machine can divide these sets of images into different categories based on the similarities between images. Machine learning depends on two strategies named regression and classification. Regression is a forecasting strategy utilized for continuous variables. On other hand, the classification predicts the events of distinct outputs, for instance, it can predict the day status as be sunny or foggy. For example, the linear regression technique can be used to forecast continuous variables, whilst the discrete variables can be predicted by utilizing the logistic regression technique. There are a lot of techniques utilized for machine learning, such as neural networks and random forests. Among these techniques, the decision tree is an effective technique that can perform both regression and classification. Furthermore, it can be utilized for the prediction of both continuous and discrete outputs. The following subsection discusses the decision tree in more detail.

### Decision Tree

The decision tree is an algorithm to generate appropriate rules in order to approximate the discrete functions. This algorithm analyzes and classifies the input data then it can make a decision for the new data. The learning algorithm discovers the rules between the input data to build the decision tree [27]. The decision tree with high accuracy and small scale is the main target of the decision tree algorithm. The classification in the decision tree algorithm is carried out as sets of “if–then” or a conditional distributed probability based on the class and features space. The decision algorithm has three stages: selection of features, generation of decision, and pruning. The processing in the decision algorithm, initiates from the root node by testing, for instance, a certain feature, then this feature can be assigned for the next node according to the results of the last testing. The values of the tested feature are divided for each child node simultaneously. The testing and assignment of the feature are still carried out until reaching the leaf node. In the final stage, the feature values are divided into the leaf node class. The decision tree applies an index named information entropy to detect the uncertainty of the tested set and utilizes the information gain as a measure of the uncertainty or purity. Then, it can split the node based on the feature that has the largest information gain. The entropy utilizes the expected information to detect if the set needs to be divided into multiple classes or not [28]. The information index of symbol *x_i_* is formulated as follows:

(1) $$I(x_{i})=-\log_{2}P(x_{i})$$

where *P*(*x_i_*) represents the probability of the selected category.

The entropy can be calculated by adding the information values of all categories as follows:

(2) $$E=-\sum_{i=1}^{m}P(x_{i})\log_{2}P(x_{i})$$

where *m* is the total number of variables that require classification. The greater entropy indicates the greater uncertainty of the variable. After the calculation of the entropy probability of the estimated data, the empirical entropy that corresponds to this probability is formulated as follows:

(3) $$E(Y)=-\sum_{i=1}^{c}\frac{\left|n_{i}\right|}{\left|M\right|}\log_{2}\frac{\left|n_{i}\right|}{\left|M\right|}$$

where *c* is the maximum integer limit of the dataset *Y*, *n_i_* is the size of *Y*, and $M=\sum_{i=1}^{c}n_{i}$. Then, the uncertainty of variable *Y* under the information of known variable *X* can be represented by the conditioned entropy as follows [28]:

(4) $$E(Y|X)=-\sum_{i=1}^{m}p_{i}E(Y|X=x_{i})$$

The entropy is utilized to formulate the information gain. The information gain is the relative gain of the feature and it is formulated as follows:

(5) G=E(Y)−E(Y|X)

where E(Y) is the empirical entropy of the training dataset *Y*, and E(Y|X) is the conditional entropy of the feature ‘*X*’ in the dataset ‘*Y*’. The size of information gain changes according to the training dataset. When the problem is difficult, the empirical entropy and the information gain increase. On the contrary, the information gain is small when the problem is simple.

## 4. Smart Energy System and IoT Technology

**Figure 2:**
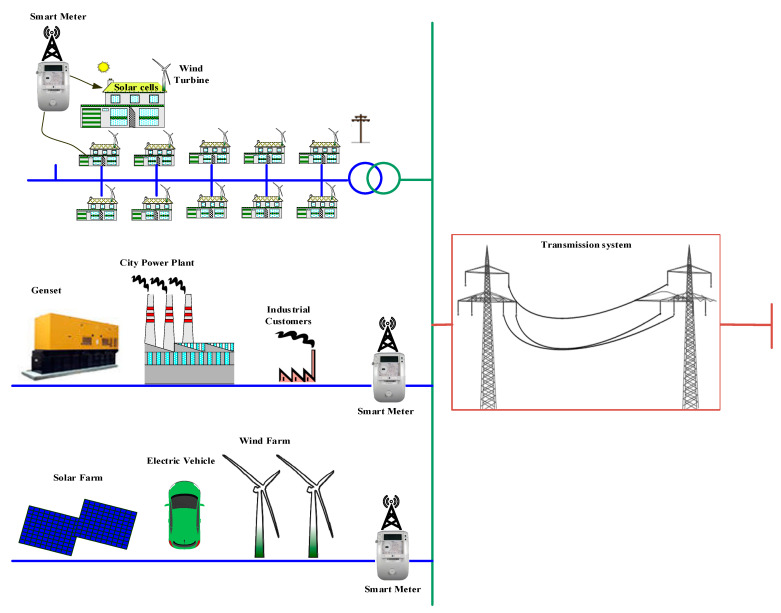
Smart grid with distributed power suppliers.

The modern power grid has a lot of challenges to cope with in the interpretation of renewable energy. This grid requires a new scenario to make it smart in order to manage the energy flow. Furthermore, the new scenario must take into account the interoperability of the old and new equipment. The new scenario has a lot of power suppliers, as shown in Figure 2, who provide their users with energy and supply the surplus to the main network. However, the discontinuity of renewable energy sources (photovoltaic and wind generation systems) increases the risk of the blackout to the whole network. So, the new power grid requires smart sensing about the variation of energy to cope with the sudden disconnection and the penetration of renewable energy resources.

The smart meter is an electronic device that can communicate the measuring information of power, gas, and water consumption for monitoring, control, and billing. The smart meter can send the reading to the utility company directly. Furthermore, it provides consumers with all details about their consumption and costs. This information allows the power grid to manage the fault events and the rapid changes in energy. The power grid can be involved with IoT that can provide the smart grid with a lot of opportunities for sharing the information into the whole power grid. The IoT allows sensors to share data that enhance the management of the energy in the whole grid. Furthermore, the new sensing system provides the grid with a lot of features and flexibility to monitor, predict, and control consumption. Reliability and security are the main challenges faced by the sharing of data through IoT. 

This paper introduces a new reliable intelligent system to check the sharing data based on the decision tree to classify the real data and fake data. Furthermore, the algorithm can check the amount of data loss due to the low speed and discontinuity of the internet. This intelligent system enhances the decision of the power grid about energy management and control.

The novelty of this work is to introduce a new infrastructure based on the decision tree technique to analyze, monitor, and secure the output data of the smart meters, thereby investigating if this data is real data or fake (meaning hacking). This feature allows compensating the industrial environment affects on the efficiency of the meters (e.g., temperature, humidity, and noise signals). Most importantly, the proposed infrastructure validates the amount of data loss via communication channels and the internet connection, providing an option to measure the internet speed and status and minimize the system cost. The decision tree is used here as an effective machine learning tool to perform both regression and classification for the meters’ data. The main advantage of the decision tree is its simplicity to build and intuition to understand. However, its limitation is that it requires suitable depth to perform with good accuracy. Note that data monitoring is conducted based on the industrial digital twins’ framework. The proposed infrastructure can provide a reliable and effective industrial decision to enhance the investments in industry 4.0.

## 5. Results and Discussion

In this section, the decision tree is devoted to classifying the data of the smart meter. Then, the model created by the decision tree is encrypted with the smart system to provide online validation for the meter output via IoT. A real-time dataset is collected from the smart meter at different operation conditions for the training and testing of the decision tree. A fake dataset is added to the real-time dataset of the smart meter. The real data of the smart meter is labeled by 1 and the fake data is labeled by 2 in order to train and test the decision tree. The dataset is divided into training data by 70% and testing data by 30%. Table 1 shows a few samples of the dataset that are utilized to train and test the decision tree. The measured current ‘I’ and its rate of change ‘Delta I’ are utilized as the inputs of the decision tree. The Label of the real data and fake data is used as the output of the decision tree.

**Figure 3:**
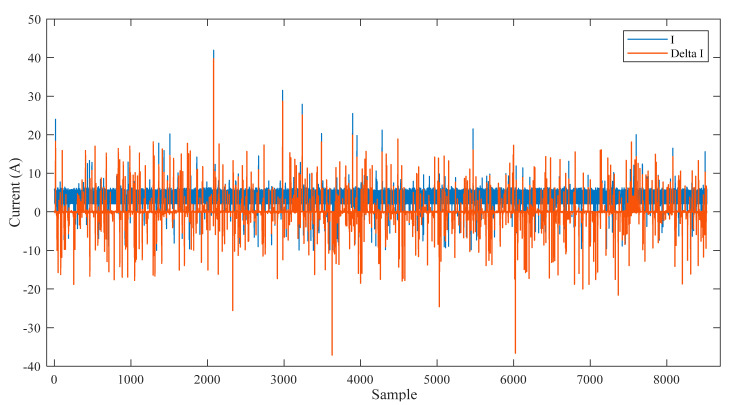
The input training data of the decision tree.

**Figure 4:**
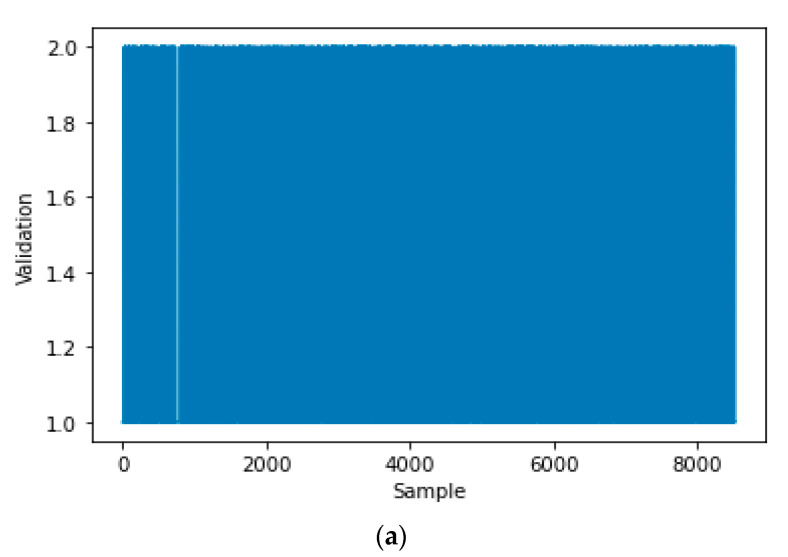

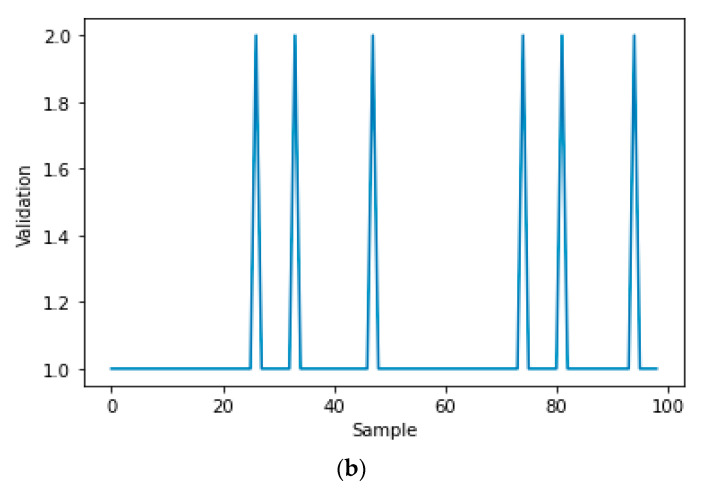
The output training dataset of the decision tree: (**a**) all output of training dataset, (**b**) zoomed in on a few samples from the output training dataset.

**Figure 5:**
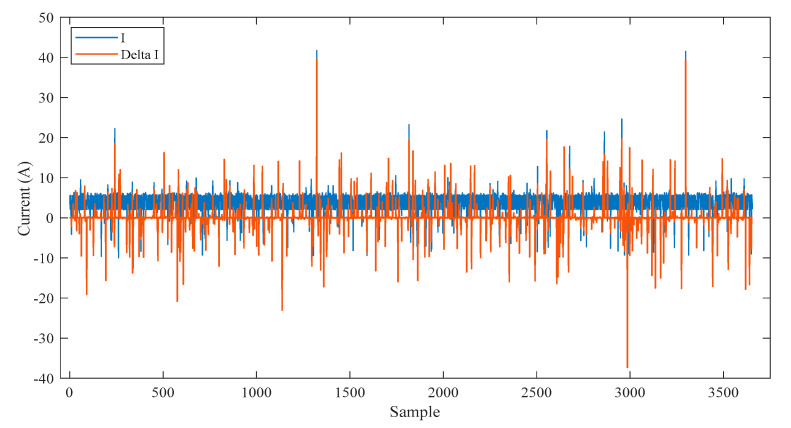
The input testing data of the decision tree.

**Figure 6:**
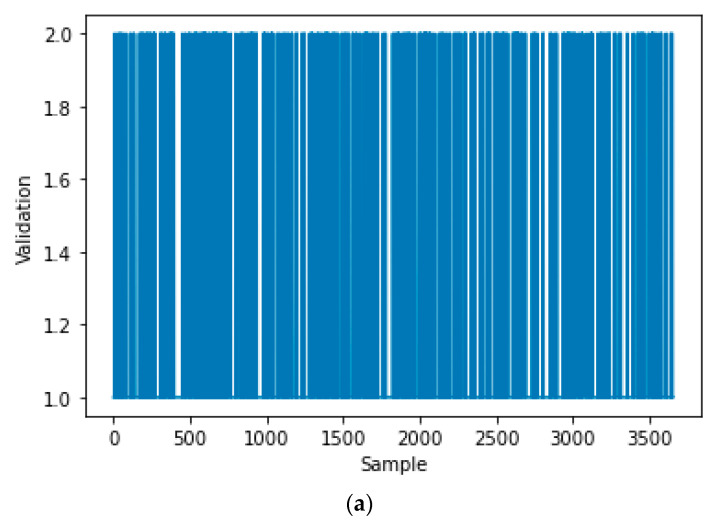

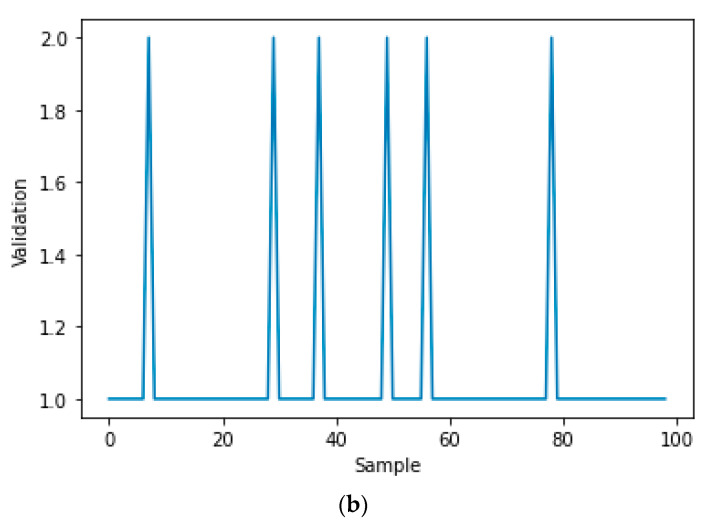
The output testing dataset of the decision tree: (**a**) all output of testing dataset, (**b**) zoomed in on a few samples from the output testing dataset.

**Figure 7:**
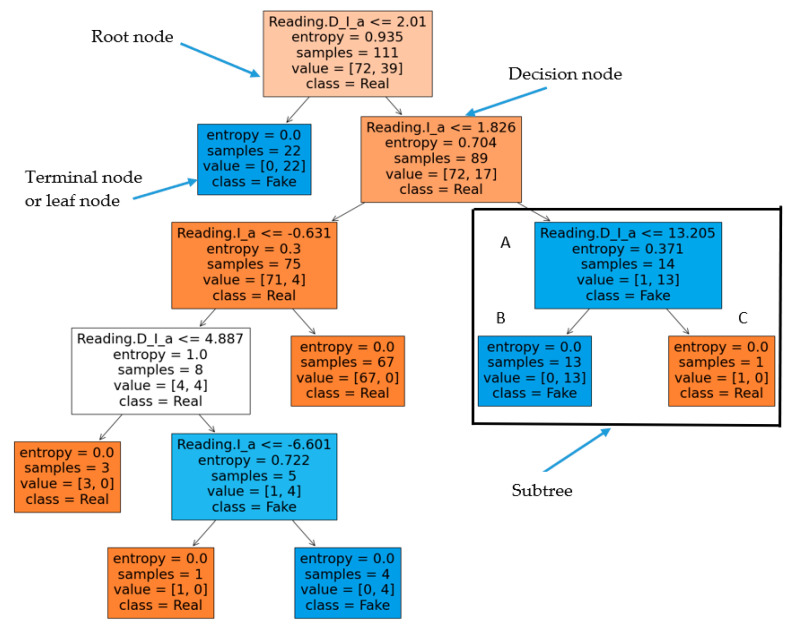
Identification of smart meter reading into real and fake data by decision tree algorithm.

Figure 3 shows all samples of the inputs training dataset, that is represented by the current ‘I’ and its rate of change ‘Delta I’. Figure 4a shows all samples of the output training dataset, that is represented by the labeled validation (Real ‘1′, Fake ‘2′). Figure 4b is a zoomed in image of Figure 4a for a few samples of the output training dataset. All samples of the input testing dataset are clear in Figure 5, while all samples of the output testing dataset are shown in Figure 6a. Further, Figure 6b shows a zoomed in image for a few samples of the output testing dataset from Figure 6a. The process of the decision tree is described in Figure 7 and it includes the following components:

- An entire population is represented by the “root node”.
- The “splitting” is the step of dividing the node into two or more sub-nodes.
- The “decision node” is represented by a sub-node that is split into different sub-nodes.
- The final node in a decision tree is called “leaf/terminal node”.
- The process of removing sub-nodes from the decision node named “Pruning”, this process is unlike splitting.
- The branch is a subsection of the entire tree and it is named “Sub-tree”.
- In the sub-tree, the node that split into two nodes named the parent as “A” in Figure 7 and new nodes named children as “B” and “C” in Figure 7.

Table 2 shows how the decision tree algorithm determines the uncertainty, named entropy, according to the following formula [29]:

(6) $$-(p\log_{2}p+q\log_{2}q)$$

where *p* represents the probability of real data happening, and *q* represents the probability of fake data happening. Reference [30] compares the accuracy of the decision tree with other machine learning techniques: (1) Artificial neural network, (2) K-nearest neighbor, (3) Logistic regression, and (4) Naïve Bayes. Notably, the decision tree performed superior accuracy in a lot of works compared with the four machine learning algorithms. For this reason, the decision tree has been assigned in this work rather than the others.

**Figure 8:**
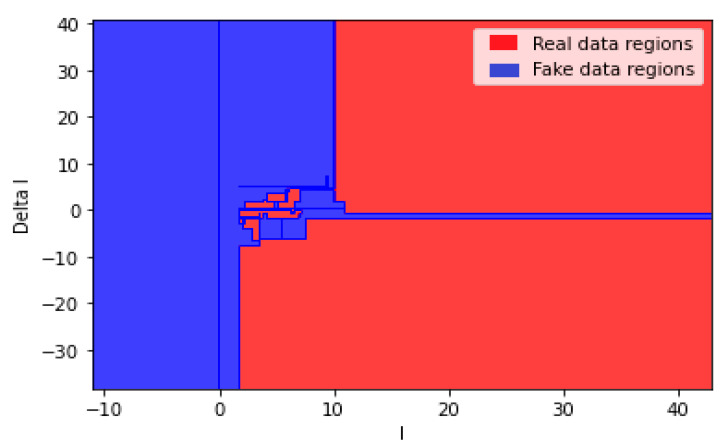
The classified regions of the real and fake data based on the decision tree.

**Figure 9:**
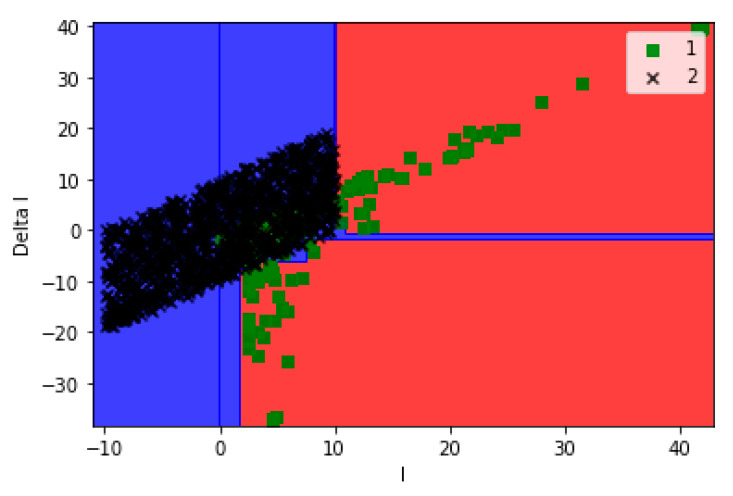
The locations of the testing dataset on the regions of the real and fake data.

Figure 8 shows the output classification regions of the decision tree after training, while Figure 9 shows the locations of the testing data in the classified regions of real and fake data.

After training and testing, the created model of the decision tree is encrypted with the IoT system to classify the online reading of the smart meter and publish it in the dashboard. Furthermore, the validation of the data loss due to the variations of the internet speed is performed during the online operation. The following pseudo-code (Algorithm 1) describes the full operation of data acquisition, validation, and visualization,
**Algorithm 1** The pseudo-code of data acquisition, validation, and visualization**1: *Read*** data from the smart meter**2: *Input*** the data to the Decision tree model**3: *Classify*** the data by the Decision tree**4:   if** the output of the Decision tree == 1**5:       *Publish*** that the data is ‘Real’**6:   else****7:       *Publish*** that the data is ‘Fake’**8:   end if****9: *Record*** the receiving time of data**10: *Calculate*** the change of time**11:   if** the change of time ≤ sample rate of the smart meter**12:       *Publish*** ‘No loss data’**13:   else****14:       *Publish*** ‘There is loss data’**15:   end if****16: *Publish*** the reading of the smart meter

The final results about the data validation and data loss due to the internet problems will be recorded on the database server and presented on the dashboard of the IoT platform.

### 5.1. Scenario 1: Normal Case

**Figure 10:**
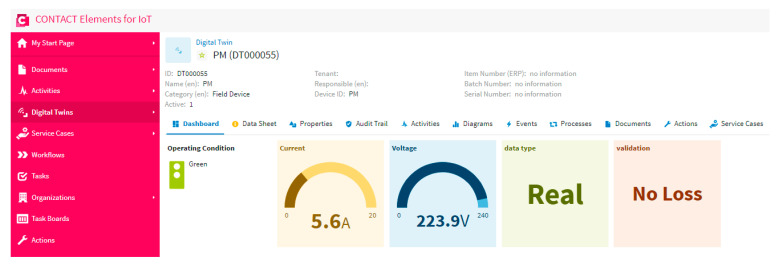
The dashboard of the IoT platform results in the case of scenario 1.

In this scenario, the proposed system is tested when the data of the meter is real and the internet network does not overload. Figure 10 shows the output of the IoT system due to this test, that is presented in the dashboard of the IoT platform. As is clear in this figure, the data is real and there is no loss. Furthermore, the operation condition is green, as is clear in the traffic light, which means that the system is stable, and so no event and/or alarms are noticed. This proves that the model of the decision tree works well without an error.

### 5.2. Scenario 2: Testing Fake Data and No Loss

**Figure 11:**
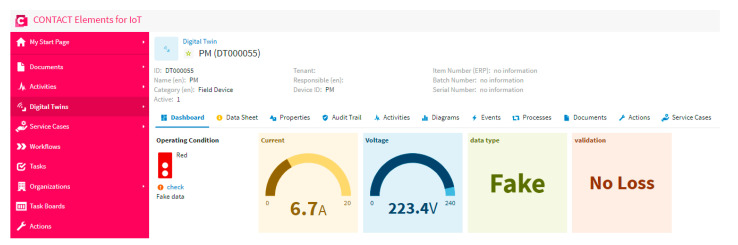
The dashboard of the IoT platform results in a case of fake data and no loss testing.

In this scenario, the proposed system is tested when the data of the meter is fake and the internet network does not overload. As is clear in Figure 11, the data is fake and there is no loss. This proves that the model of the decision tree work can catch the fake data to help the user to secure and check the smart meter. Furthermore, the fake data and the corresponding time are recorded in the database for any future action and forecasting. Besides, the traffic light changed to a red color to hint the user about the abnormal case of fake data. Moreover, the dashboard shows that there is no loss in the entry data, which proves that the internet network is stable and works effectively without overload.

### 5.3. Scenario 3: Testing Internet Capability 

**Figure 12:**
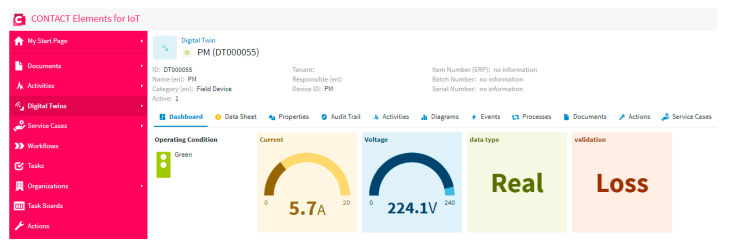
The dashboard of the IoT platform results in the case of testing internet capability.

Internet speed represents a big challenge against the IoT systems. So, the smart system must check the data loss and the required internet speed for the network. In this scenario, the proposed system is tested to check the data loss and publish the results on the dashboard of the IoT platform. Figure 12 shows that there is data loss, as shown in the dashboard of the IoT platform. This concludes that the network speed is not enough for the smart system. In spite of the fact that the data is real and secured, as is clear in the dashboard, there is not enough criteria to make a good decision due to the presence of losses in the data. This data loss causes a high temperature, which can damage the electrical machine.

**Figure 13:**
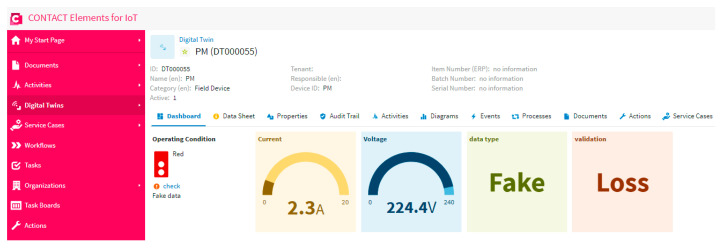
The dashboard of the IoT platform results in a case of testing the fake data and internet capability.

This case is carried out to check both the data type and internet capability together. Figure 13 shows that the data is fake and there is data loss, as shown in the dashboard of the IoT platform. This is the most abnormal case and it indicates that the data is fake and the network speed is not enough for the smart system. Furthermore, when the operation condition is a red color, as shown in the traffic light, the system is unstable, resulting in abnormal events and/or alarms.

## 6. Conclusions

This work introduced a newly developed intelligent technique to check the reliability of the IoT smart systems. The data validation of the smart meter is accomplished by a machine learning technique named decision tree. The decision tree technique performed the regression and classification of the smart meter reading to real and fake types. Furthermore, the developed algorithm can detect data loss due to unstable internet signals. The online output of the system, like data loss, real, and fake data, are presented in the dashboard of the IoT platform. The efficacy of the proposed infrastructure has been evaluated by three scenarios. Scenario 1 proves that the data is real and there is no loss, while the model of the decision tree works well without an error. Regarding Scenario 2, it shows that the model of the decision tree can catch the fake data to help the user to secure and check the smart meter. Accordingly, the traffic light has been changed to a red color to hint the user about the abnormal case of fake data. In the final scenario, the proposed infrastructure has been tested to check the data loss and publish the results on the dashboard of the IoT platform, where it is concluded that the network speed is not enough for the smart system. Generally, the proposed method enhances the reliability of the smart IoT systems, which increases the investments in industry 4.0. Besides, it can be applied to different kinds of sensors and machines in future work.

## Figures and Tables

**Table 1:** Samples of training and testing data of the decision tree.

Current ‘I’	Change in Current ‘Delta I’	Validation	Label of Validation Real ‘1′, Fake ‘2′
5.7	0	Real	1
2.7	−3	Real	1
2.8	0.1	Real	1
2.7	−0.1	Real	1
2.8	0.1	Real	1
2.8	0	Real	1
−6.3	−9.1	Fake	2
5.6	11.9	Fake	2
−6.1	−11.7	Fake	2
9.8	15.9	Fake	2
6	−3.8	Fake	2
−1.6	−7.6	Fake	2

**Table 2:** Sample of decision tree selection based on the probability of events.

Scenario	Real Data Uncertainty	Fake Data Uncertainty	Overall Uncertainty
Equal chances of win	−0.5 log_2_ (0.5) = 0.5	−0.5 log_2_ (0.5) = 0.5	0.5 + 0.5 = 1
80% chances of a win for Real data	−0.8 log_2_ (0.8) = 0.2575	−0.2 log_2_ (0.2) = 0.4644	0.2575 + 0.4644 = 0.7219

## Data Availability

The data presented in this study are available on request from the corresponding author.

## References

[1] Lin J.C.-W., Yeh K.-H. (2020). Security and Privacy Techniques in IoT Environment. Sensors.

[2] Lloret J., Tomas J., Canovas A., Parra L. (2016). An Integrated IoT Architecture for Smart Metering. IEEE Commun. Mag..

[3] Alablani I., Alenazi M. (2020). EDTD-SC: An IoT Sensor Deployment Strategy for Smart Cities. Sensors.

[4] Bedi G., Venayagamoorthy G.K., Singh R., Brooks R.R., Wang K.C. (2018). Review of Internet of Things (IoT) in Electric Power and Energy Systems. IEEE Internet Things J..

[5] Zhao L., Brandao Machado Matsuo I., Zhou Y., Lee W.J. (2019). Design of an Industrial IoT-Based Monitoring System for Power Substations. IEEE Trans. Ind. Appl..

[6] Morello R., De Capua C., Fulco G., Mukhopadhyay S.C. (2017). A smart power meter to monitor energy flow in smart grids: The role of advanced sensing and iot in the electric grid of the future. IEEE Sens. J..

[7] Trakadas P., Simoens P., Gkonis P., Sarakis L., Angelopoulos A., Ramallo-González A.P., Skarmeta A., Trochoutsos C., Calvο D., Pariente T. (2020). An Artificial Intelligence-Based Collaboration Approach in Industrial IoT Manufacturing: Key Concepts, Architectural Extensions and Potential Applications. Sensors.

[8] Petrillo A., Picariello A., Santini S., Scarciello B., Sperlí G. (2020). Model-based vehicular prognostics framework using Big Data architecture. Comput. Ind..

[9] De santo A., Galli A., Gravina M., Moscato V., Sperli G. (2020). Deep Learning for HDD health assessment: An application based on LSTM. IEEE Trans. Comput..

[10] Chang C.-Y., Kuo C.-H., Chen J.-C., Wang T.-C. (2015). Design and Implementation of an IoT Access Point for Smart Home. Appl. Sci..

[11] Abate F., Carratù M., Liguori C., Paciello V. (2019). A low cost smart power meter for IoT. Meas. J. Int. Meas. Confed..

[12] García-Magariño I., Nasralla M.M., Nazir S. (2020). Real-Time Analysis of Online Sources for Supporting Business Intelligence Illustrated with Bitcoin Investments and IoT Smart-Meter Sensors in Smart Cities. Electronics.

[13] Chen Y., Martínez J.-F., Castillejo P., López L. (2017). An Anonymous Authentication and Key Establish Scheme for Smart Grid: FAuth. Energies.

[14] Nabil M., Ismail M., Mahmoud M.M.E.A., Alasmary W., Serpedin E. (2019). PPETD: Privacy-Preserving Electricity Theft Detection Scheme with Load Monitoring and Billing for AMI Networks. IEEE Access.

[15] Cunha V.C., Freitas W., Trindade F.C.L., Santoso S. (2020). Automated Determination of Topology and Line Parameters in Low Voltage Systems Using Smart Meters Measurements. IEEE Trans. Smart Grid.

[16] Ferreira T.S.D., Trindade F.C.L., Vieira J.C.M. (2020). Load Flow-Based Method for Nontechnical Electrical Loss Detection and Location in Distribution Systems Using Smart Meters. IEEE Trans. Power Syst..

[17] Bu F., Dehghanpour K., Yuan Y., Wang Z., Zhang Y. (2020). A Data-Driven Game-Theoretic Approach for Behind-the-Meter PV Generation Disaggregation. IEEE Trans. Power Syst..

[18] Wang Y., Chen Q., Hong T., Kang C. (2019). Review of Smart Meter Data Analytics: Applications, Methodologies, and Challenges. IEEE Trans. Smart Grid.

[19] Rahman M.A., Manshaei M.H., Al-Shaer E., Shehab M. (2017). Secure and private data aggregation for energy consumption scheduling in smart grids. IEEE Trans. Dependable Secur. Comput..

[20] Ibrahem M.I., Nabil M., Fouda M.M., Mahmoud M., Alasmary W., Alsolami F. (2020). Efficient Privacy-Preserving Electricity Theft Detection with Dynamic Billing and Load Monitoring for AMI Networks. arXiv.

[21] Kumar P., Lin Y., Bai G., Paverd A., Dong J.S., Martin A. (2019). Smart Grid Metering Networks: A Survey on Security, Privacy and Open Research Issues. IEEE Commun. Surv. Tutor..

[22] Sun Q., Li H., Ma Z., Wang C., Campillo J., Zhang Q., Wallin F., Guo J. (2016). A Comprehensive Review of Smart Energy Meters in Intelligent Energy Networks. IEEE Internet Things J..

[23] Spanò E., Niccolini L., Di Pascoli S., Iannaccone G. (2015). Last-meter smart grid embedded in an internet-of-things platform. IEEE Trans. Smart Grid.

[24] Kabugo J.C., Jämsä-Jounela S.L., Schiemann R., Binder C. (2020). Industry 4.0 based process data analytics platform: A waste-to-energy plant case study. Int. J. Electr. Power Energy Syst..

[25] Aheleroff S., Xu X., Lu Y., Aristizabal M., Pablo Velásquez J., Joa B., Valencia Y. (2020). IoT-enabled smart appliances under industry 4.0: A case study. Adv. Eng. Inform..

[26] IoT Platform for Digital Business Models|CONTACT Software. https://www.contact-software.com/en/products/iot-platform-for-digital-business-models/?fbclid=IwAR0oYDd4qHpCd0BEZaGrLHEAQGYoQ2BhBmDzbF35-cyM6QrNHAkziWDC8yo.

[27] Myles A.J., Feudale R.N., Liu Y., Woody N.A., Brown S.D. (2004). An introduction to decision tree modeling. J. Chemom..

[28] Liu S., Yang Z., Li Y., Wang S. (2020). Decision Tree-Based Sensitive Information Identification and Encrypted Transmission System. Entropy.

[29] Ayyadevara V.K. (2018). Pro Machine Learning Algorithms.

[30] Uddin S., Khan A., Hossain M.E., Moni M.A. (2019). Comparing different supervised machine learning algorithms for disease prediction. BMC Med. Inform. Decis. Mak..

